# C-Reactive Protein and B-Type Natriuretic Peptide Yield Either a Non-Significant or a Modest Incremental Value to Traditional Risk Factors in Predicting Long-Term Overall Mortality in Older Adults

**DOI:** 10.1371/journal.pone.0075809

**Published:** 2013-09-25

**Authors:** Alline M. Beleigoli, Eric Boersma, Maria de Fátima H. Diniz, Pedro G. Vidigal, Maria Fernanda Lima-Costa, Antonio L. Ribeiro

**Affiliations:** 1 Department of Internal Medicine, Faculty of Medicine, Universidade Federal de Minas Gerais, Belo Horizonte, Brazil; 2 Post-Graduation Department, Faculty of Medicine, Universidade Federal de Minas Gerais, Belo Horizonte, Brazil; 3 Department of Cardiology, Erasmus Medical Center, Rotterdam, The Netherlands; 4 Department of Diagnostic Medicine, Faculty of Medicine, Universidade Federal de Minas Gerais, Belo Horizonte, Brazil; 5 Centro de Pesquisas René Rachou, Fundação Oswaldo Cruz, Belo Horizonte, Brazil; 6 Hospital das Clínicas, Universidade Federal de Minas Gerais, Belo Horizonte, Brazil; Innsbruck Medical University, Austria

## Abstract

**Background:**

New biomarkers may aid in preventive and end-of-life decisions in older adults if they enhance the prognostic ability of traditional risk factors. We investigated whether C-reactive protein (CRP) and/or B-type natriuretic peptide (BNP) improve the ability to predict overall mortality among the elderly of the Bambuí, Brazil Study of Aging when added to traditional risk factors.

**Methods:**

From 1997 to 2007, 1,470 community-dwelling individuals (≥60 years) were followed-up. Death was ascertained by continuous verification of death certificates. We calculated hazard ratios per 1 standard deviation change (HR) of death for traditional risk factors only (old model), and traditional risk factors plus CRP and/or BNP (new models) and assessed calibration of the models. Subsequently, we compared c-statistic of each of the new models to the old one, and calculated integrated discriminative improvement (IDI) and net reclassification improvement (NRI).

**Results:**

544 (37.0%) participants died in a mean follow-up time of 9.0 years. CRP (HR 1.28, 95% CI 1.17-1.40), BNP (HR 1.31 95% CI 1.19-1.45), and CRP plus BNP (HR 1.26, 95% CI 1.15-1.38, and HR 1.29, 95% CI 1.16-1.42, respectively) were independent determinants of mortality. All models were well-calibrated. Discrimination was similar among the old (c-statistic 0.78 [0.78-0.81]) and new models (p=0.43 for CRP; p=0.57 for BNP; and p=0.31 for CRP plus BNP). Compared to the old model, CRP, BNP, and CRP plus BNP models led to an IDI of 0.009 (p<0.001), -0.005 (p<0.001) and -0.003 (p=0.84), and a NRI of 0.04 (p=0.24), 0.07 (p=0.08) and 0.06 (p=0.10), respectively.

**Conclusions:**

Despite being independent predictors of long-term risk of death, compared to traditional risk factors CRP and/or BNP led to either a modest or non-significant improvement in the ability of predicting all-cause mortality in older adults.

## Introduction

Assessing the risk of mortality by clinical prediction scores is particularly interesting in the elderly, as they may aid in critically planning and carrying out preventive and end-of-life quality interventions in individuals aged ≥60 years who comprised approximately 51% of deaths worldwide [1] and 59% of total deaths in Brazil [2]. However, it has been described that traditional risk factors tend to lose their predictive ability in this age group [3]. This leads to a special interest in the use of biomarkers, such as C-reactive protein (CRP) and B-type natriuretic peptide (BNP) in older adults.

CRP is a non-specific acute phase protein which is produced predominantly by hepatocytes under the influence of cytokines, such as interleukin (IL)-6 and tumor necrosis factor (TNF)-alpha. High sensitive (hs)-CRP has been recognized as an independent determinant of cardiovascular diseases and death in the elderly [4,5]. Additionally, it allows the recognition of a low-grade inflammatory state, which underlies both the ageing process and several conditions closely associated with ageing, such as atherosclerosis, Alzheimer’s disease, Parkinson’s disease, type 2 diabetes mellitus, sarcopenia, osteoporosis, cognitive decline, and frailty [4].

BNP, a 32-member of the family of the natriuretic peptides (NPs), is mainly secreted by ventricular cardiomyocytes, and promotes natriuresis, diuresis, vasodilatation and inhibition of the renin-angiotensin-aldosterone system [6]. Besides the hemodynamic actions, NP have anti-inflammatory, anti-fibrotic, anti-proliferative and metabolic effects [7]. Clinically, NPs have been used in the diagnosis of patients with acute dyspnea, as well as prognostic tools in conditions, such as HF [8] and acute coronary syndrome (ACS) [9]. In populations of community-dwelling elderly, NPs were independent predictors of cardiovascular events [10,11], hospitalization [12] and death regardless of previous history of cardiovascular disorders [10,11,13].

We investigated the additional value of CRP and/or BNP to traditional risk factors in the prediction of 11-year all-cause mortality risk in the population of elderly of the Bambuí, Brazil Cohort Study of Aging (BHAS).

## Methods

### Ethics Statement

All participants signed an informed consent form and authorized death certificate verification. The BHAS was approved by the ethics board of the Fundação Oswaldo Cruz, Belo Horizonte, Brazil.

### Study design and population

The study was conducted in the Bambuí City (approximately 15,000 inhabitants), located in the state of Minas Gerais, southeast region of Brazil. Procedures used in the BHAS have been described in detail elsewhere [14]. Briefly, the baseline cohort population comprised 1,606 (92.2%) of all residents (1,742) aged 60 years or more on January 1^st^, 1997, who were identified by means of a complete census. Baseline data collection (standardized interviews, blood tests, blood pressure measurements, and electrocardiograms) was performed from February to May 1997.

### Outcome Ascertainment

Deaths assigned to any cause and reported by next of kin during the annual follow-up interview and ascertained through the Brazilian Mortality Information System (Sistema de Informações sobre Mortalidade) with the permission of the Ministry of Health from study enrolment to December 31^st^, 2007 were included in this analysis. Death certificates were obtained for 98.9% of all deceased participants.

### CRP

Data on hs-CRP was available in 1,470 (91.5%) participants. Blood samples were collected after 12-hours fast and serum samples were stored at -80°C. Measurements were taken by the CRP immunonephelometric method, Dade-Behring N Latex CRP particle-enhanced immunoassay on an automatic nephelometer (BNII™,Dade Behring, Marburg, Germany) traceable to the international reference standard CRM 470 [15]. The limit of detection of the CRP assay as provided by the manufacturer is 0.175-500mg/L and the CV is 2.2-5.8%. Although very high (≥10mg/L) hs-CRP values are likely to be due to systemic inflammatory states such as major infection, trauma, or chronic inflammatory disease, we decided not to exclude them as they might also be related to cardiovascular risk and/or death [16].

### BNP

BNP was measured in blood samples collected in tubes containing ethylenediaminetetraacetic acid and stored at -80°C until used. Subjects were asked to fast for 12 hours prior to early-morning (6:30–8:30 AM) phlebotomy. A microparticle-based immunoassay (MEIA/AxSYM; Abbott Laboratories) with 15-5,000 pg/mL as limits of detection and average interassay coefficients of variation of 12% was used.

### Traditional risk factors

The choice of the traditional risk factors to enter the models was based on results of population studies about the risk of death associated with non-communicable diseases, as well as in previous investigations in the BHAS [17,18,19,20,21]. Current smoking was ascertained by the standardized BHAS questionnaire. Systolic blood pressure was defined as the mean of two lowest out of three standardized measurements. Fasting blood glucose, total cholesterol (TC) and HDL were determined using an automated analyser (Eclipse Vitalab, Merck, The Netherlands). Diabetes was defined as a 12-h-fast glucose ≥126 mg/dL and/or the use of insulin or oral hypoglycemic agents. Two high-precision digital scales (range 0–150 kg×0.1 kg) were used for the measurement of weight (kg) and height (cm). BMI was calculated as weight (kg)/ height (m)^2^. Waist circumference (WC; cm) was measured at umbilicus height by inelastic tapes. Leisure physical activity was verified by standardized interview and defined as walking and/or practicing any other physical exercise for at least 3-5 days/week, 20-30 min/day within the last 90 days. Chagas disease was also investigated as a traditional risk factor because it used to be endemic in the region of Bambuí city until the 1970s. Following the control by insecticides, the incidence of the infection with *Trypanossomacruzi* has dramatically decreased, but a cohort effect led to a high prevalence (38.1%) of chronic Chagas disease in the elderly [22]. Diagnosis relies on serologic methods and was defined by seropositivity in all three of the different parallel tests: a hemagglutination assay (Biolab Mérieux SA, Rio de Janeiro, Brazil) and 2 enzyme-linked immunosorbent assays (Abbott Laboratories, Inc., North Chicago, Illinois; and Wiener Laboratories, Rosario, Argentina). The absence of infection was defined as consistent seronegativity. The agreement (Cohen’s kappa) among these assays was 0.989 (p < 0.001).

### Statistical analysis

Normal distribution of continuous data was verified by histograms and Komolgorov-Smirnov tests. Variables with a skewed distribution were log-transformed (BNP, CRP). Continuous variables were described by mean and standard deviation (SD), or median and inter-quartile range (IQR). To compare baseline characteristics across BNP and CRP tertiles we used the Chi-square test for linear trends, the ANOVA with *post hoc* Bonferroni correction for continuous variables with normal distribution (age, systolic blood pressure, total cholesterol, HDL, and WC) and the Mann-Whitney tests. We compared survival rates across the tertiles of CRP (<1.88, 1.88-5.18, ≥5.18 mg/L) and BNP (<55, 55-119, ≥119 pg/mL) by means of Kaplan Meier curves and log-rank tests. To deal with missing values, we performed multiple imputation by fitting logistic and linear regression models with both the predictors and the outcome, as well as other variables regarded as important to explain the missing values [23]. This procedure generated five complete datasets, which were used to estimate crude and adjusted hazard ratios (HR) and 95% confidence intervals (CI) for death by Cox regression models. Proportional hazards assumption was verified by plotting the log of the cumulative hazard against the log of follow-up time. The first model (old model) was fitted for traditional risk factors only: age (continuous), gender, current smoking (no, yes), systolic blood pressure (continuous), total cholesterol (continuous), HDL (continuous), BMI (continuous and squared), WC (continuous), physical activity (no, yes), and Chagas disease (no, yes). For the other three models (new models), we additionally adjusted for log-transformed CRP (continuous) and log-transformed BNP (continuous) individually and simultaneously. As Chagas disease is a prevalent comorbidity in our population, we tested for the effect modification of Chagas on the association between each of the biomarkers and death by means of product terms of interaction. We also performed sensitivity analysis with complete original data.

To aid in clinical decision-making in the context of the rational use of resources, a biomarker must be both an independent determinant of the outcome and incrementally improve the predictive ability of models that are based on established risk factors only [16]. Thus, we also assessed the performance of the models by calibration and discrimination. For assessment of calibration, i.e. agreement between observed and predicted events, we used a modified Hosmer-Lemeshow chi-square statistic. Observed incidence of the outcome was obtained by the Kaplan-Meier estimator, which was then compared to the probabilities of events predicted by Cox models at the end of the follow-up (t=11), across ten groups yielded by the deciles of the predicted probabilities [24]. For assessment of the ability of each survival model to discriminate between individuals at different risk levels of death, we used Harrell’s C statistics and compared the ROC for each new model to the old one by means of the Hanley and McNeill [25] [26]. Additionally, models with CRP and BNP individually and in combination were each compared to the model with traditional risk factors only by means of integrated discrimination improvement (IDI) and retrospective category-free net reclassification improvement (NRI). IDI refers to differences in integrated sensitivity and integrated one minus specificity between the new and the old models [27]. Category-free NRI, according to Pencina et al., quantifies how the addition of a new biomarker correctly increases (upwards movements) or decreases (downwards movements) the risk predicted by the model for events and non-events. NRI calculations were based on the predicted probabilities of an event derived from predicted probabilities at the end of the follow-up (t=11) estimated by Cox regression models [28]. All the p values given are two-sided with the level of significance set to p<0.05, except for multiple comparisons when it was set to 0.05 divided by the number of comparisons. We used SPSS for Windows 17.0 and R 2.15.1 (package survivalROC).

## Results

Mean age (SD) of the participants was 69.1 (7.2) years and 895 (61.0%) were female. Mean follow-up time was 9.0 years. At 11-years of follow-up, 544 participants (37.0%) died and 89 (5.5%) were lost to follow-up, leading to 13,230 person-years of observation. Subjects with complete follow-up had similar median CRP (3.25 mg/L, IQR 1.45-6.70 versus 3.06 mg/L, IQR 1.26-5.73; p=0.38) and BNP values (82 pg/mL, IQR 44-148 versus 64 pg/mL, IQR 39-157; p=0.49) than those who were lost to follow-up.

### Characteristics

Among the traditional risk factors, BMI, diabetes, HDL and total cholesterol were different across CRP categories, and BMI and WC were different across the BNP categories (Table 1). All the traditional risk factors were independent determinants of death at 10-year follow-up in the BHAS population, except for HDL-cholesterol (Table 2).

**Table 1:** Characteristics of overall participants at baseline, and comparison according to the tertiles of C-reactive protein and B-type natriuretic peptides.

**Characteristics**	**Overall**	**CRP category**			p value*	**BNP category**			p value*
		**Low**	**Intermediate**	**High**		**Low**	**Intermediate**	**High**	
		(<1.88 mg/L)	(1.88-5.18mg/L)	(≥ 5.18mg/L)		(<55 pg/mL)	(55-119pg/mL)	(≥ 119pg/mL)	
Age, years	69 (7.4)	69 (7.0)	68 (7.0)	69 (7.0)	0.033	68 (6.6)	69 (7.1)	70 (7.7)	<0.001
Female sex (%)	964 (60.0)	264 (53.9)	313 (63.9)	318 (64.9)	<0.001	280 (57.6)	300 (60.6)	315 (63.9)	0.044
Smoking (%)	264 (16.4)	77 (16.0)	94 (19.5)	90 (18.9)	0.243	92 (19.3)	88 (18.1)	82 (17.1)	0.385
BMI (Kg/m^2^)	24.8 (21.7-27.9)	23.3 (20.8-26.4)	25.1 (22.7-26.1)	28.0 (22.4-29.3)	<0.001	25.8 (22.8-28.9)	24.3 (21.4-27.6)	24.0 (21.0-27.3)	<0.001
WC (cm)	91 (11.2)	88 (10.3)	91 (10.6)	94 (11.9)	<0.001	93 (11.4)	90 (10.9)	90 (10.9)	<0.001
Chagas disease (%)	557 (34.7)	180 (37.5)	190 (39.4)	176 (37.1)	0.889	105 (22.0)	167 (34.4)	274 (57.2)	<0.001
SBP (mm Hg)	137 (22.6)	135 (21.0)	139 (21.0)	138 (25.0)	0.060	137 (19.5)	136 (23.1)	139 (24.2)	0.072
Diabetes (%)	209 (13.0)	52 (10.8)	58 (12.1)	90 (19.0)	<0.001	73 (13.6)	66 (13.2)	63 (19.0)	0.351
Total cholesterol (mg/dL)	234 (49.1)	229 (48.0)	238 (48.0)	234 (51.0)	0.024	239 (51.4)	232 (47.2)	231 (48.3)	0.031
HDL (mg/dL)	49 (15)	50 (41-60)	46 (38-55)	45 (38-55)	<0.001	49 (14.9)	50 (15.6)	49 (14.6)	0.238
Anti-hypertensive medication (%)	742 (46.2)	200 (41.7)	251 (52.1)	279 (58.7)	<0.001	234 (49.1)	246 (50.7)	251 (52.4)	0.301
Physically active (%)†	329 (20.5)	106 (21.6)	106 (21.7)	99 (20.2)	0.584	129 (26.6)	92 (18.6)	92 (18.7)	0.002

**CRP** C-reactive protein; **BNP** B-type natriuretic peptide; **BMI** body mass index; **SBP** systolic blood pressure; **WC** waist circumference

Data based on the complete original dataset with the following number of participants with available observations for each covariate: CRP (n=1470), BNP (n=1474),Age (n=1606), sex (n=1606), smoking (n=1462), BMI (n=1450), WC (n=1454), Chagas (n=1462), systolic blood pressure (n=1459), diabetes (n=1458), total cholesterol (n=1461), HDL (n=1461), anti-hypertensive medication (n=1462), physical activity (n=1605). Frequencies (%), mean (standard deviation) and median (interquartile range) are displayed for categorical (female sex, smoking, Chagas disease, diabetes, anti-hypertensive medication and physically active) and continuous variables with normal (age, WC, SBP, total cholesterol, HDL) and skewed distribution (BMI) respectively

^*^p Value: ANOVA test with Bonferroni correction, Pearson’s chi-square test for linear trends and the Kruskal Wallis test for differences between means, frequencies and medians, respectively

† Leisure physical activity (walking or any other physical exercise) for at least 20-30min, ≥3-5 times/week

**Table 2:** Crude and adjusted hazard ratios (HR) per standard deviation change and 95% confidence interval (CI) of death in the model with traditional risk factors only.

**Variable**	**Crude HR (95% CI**)		HR adjusted for other traditional risk factors *(95% CI)	
	**Imputation dataset**	**Complete dataset**	**Imputation dataset**	**Complete dataset**
**Age (years**)	1.90 (1.89-1.92)	1.90 (1.89-1.92)	1.83 (1.68-2.00)	1.84 (1.82-1.85)
**Male sex**	1.45 (1.22-1.73)	1.45 (1.22-1.73)	1.34 (1.10-1.63)	1.35 (1.10-1.66)
**Total cholesterol (mg/dL**)	0.86 (0.78-0.95)	0.86 (0.78-0.95)	0.86 (0.78-0.95)	0.86 (0.78-0.95)
**HDL-cholesterol (mg/dL**)	1.06 (0.97-1.16)	1.06 (0.97-1.16)	1.00 (0.92-1.09)	1.00 (0.92-1.09)
**Diabetes †**	1.34 (1.06-1.70)	1.35 (1.06-1.70)	1.54 (1.20-1.98)	1.63 (1.26-2.11)
**Smoking †**	1.66 (1.36-2.04)	1.67 (1.36-2.04)	1.54 (1.23-1.92)	1.55 (1.23-1.96)
**Systolic blood pressure (mm Hg**)	1.23 (1.12-1.34)	1.23 (1.12-1.34)	1.17 (1.07-1.28)	1.17 (1.07-1.28)
**Physical activity †**	0.57 (0.44-0.73)	0.57 (0.44-0.73)	0.63 (0.49-0.81)	0.65 (0.50-0.84)
**Body mass index (kg/m^2^**)	0.71 (0.64-0.79)	0.73 (0.65-0.81)	0.20 (0.12-0.34)	0.20 (0.12-0.34)
**Waist circumference (cm**)	0.85 (0.85-0.86)	0.87 (0.80-0.95)	1.27 (1.06-1.51)	1.27 (0.22-7.33)
**Chagas disease †**	1.47 (1.23-1.76)	1.47 (1.23-1.76)	1.45 (1.21-1.75)	1.48 (1.22-1.79)

^*^ Model adjusted for: age (continuous), gender, body mass index (continuous and quadratic term), waist circumference (continuous), diabetes (no, yes), systolic blood pressure (no, yes), total cholesterol (continuous), HDL-cholesterol (continuous), current smoking (no, yes), physical activity (no, yes), Chagas disease (no, yes)

† The following categories were used as references (HR=1) for the categorical covariates: non-diabetic, non-smokers, sedentary, and non-infected by Chagas disease subjects

### Outcomes in relation to CRP and BNP

**Figure 1:**
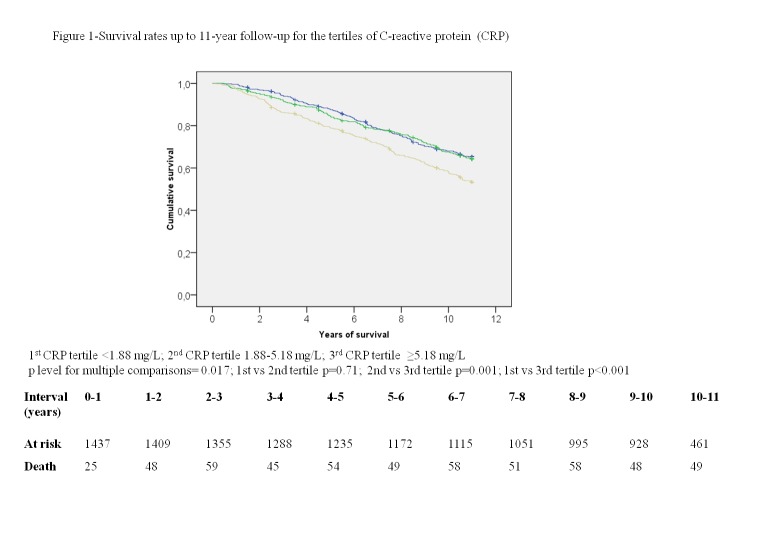
Survival rates up to 11-year follow-up for the tertiles of C-reactive protein (CRP).

**Figure 2:**
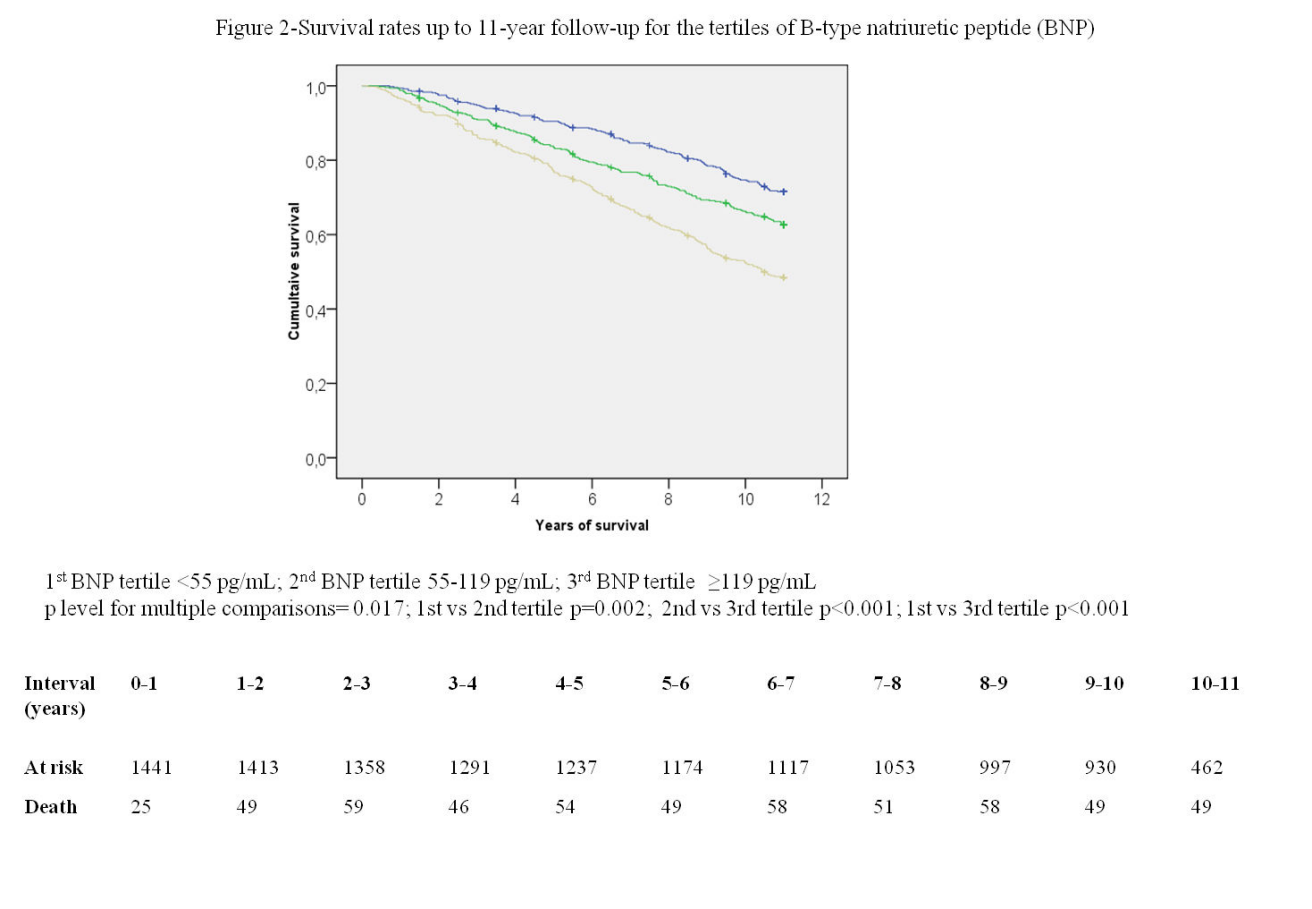
Survival rates up to 11-year follow-up for the tertiles of B-type natriuretic peptide (BNP).

Univariate survival rates at 11-year follow-up across CRP and BNP tertiles are depicted in Figures 1 and 2, respectively. After adjustment for the traditional risk factors, CRP and BNP remained independent predictors of death in the 11-year follow-up in the models in which the biomarkers were added individually (HR per 1 SD change 1.28, 95% CI 1.17-1.40, and HR per 1 SD change 1.31 95% CI 1.19-1.45, respectively) and simultaneously (HR per 1 SD change 1.26, 95% CI 1.15-1.38, and HR 1.29 95% CI 1.16-1.42, respectively). Neither was the interaction between BNP and Chagas (p=0.94), nor between CRP and Chagas (p=0.24) significant in BHAS population. These results did not change significantly from models with only complete original data in which CRP and BNP individually (HR per 1 SD change 1.24, 95% CI 1.12-1.36, and HR per 1 SD change 1.29 95% CI 1.17-1.43, respectively) and in combination (HR per 1 SD change 1.21, 95% CI 1.10-1.34, and HR per 1 SD change 1.27, 95% CI 1.15-1.41, respectively) were associated with an increased risk of death.

### Performance of the models

The deciles of observed rates of events were not significantly different from those predicted by the model with traditional risk factors only, which indicates that it was well-calibrated (X^2^=1.21, p=1.00). Models with CRP (X^2^=1.14, p=1.00) and BNP individually (X^2^=1.33, p=1.00) or in combination (X^2^=1.52, p=1.00) were also well-calibrated.

Harrell’s C-statistic for the model with traditional risk factors only was 0.78 (95% CI 0.77-0.81) and did not differ significantly from the model with CRP 0.79 (95% CI 0.77-0.81; p=0.43), BNP 0.79 (95% CI 0.76-0.81; p=0.57), and CRP and BNP simultaneously 0.79 (95% CI 0.78-0.82; p=0.31).

**Figure 3:**
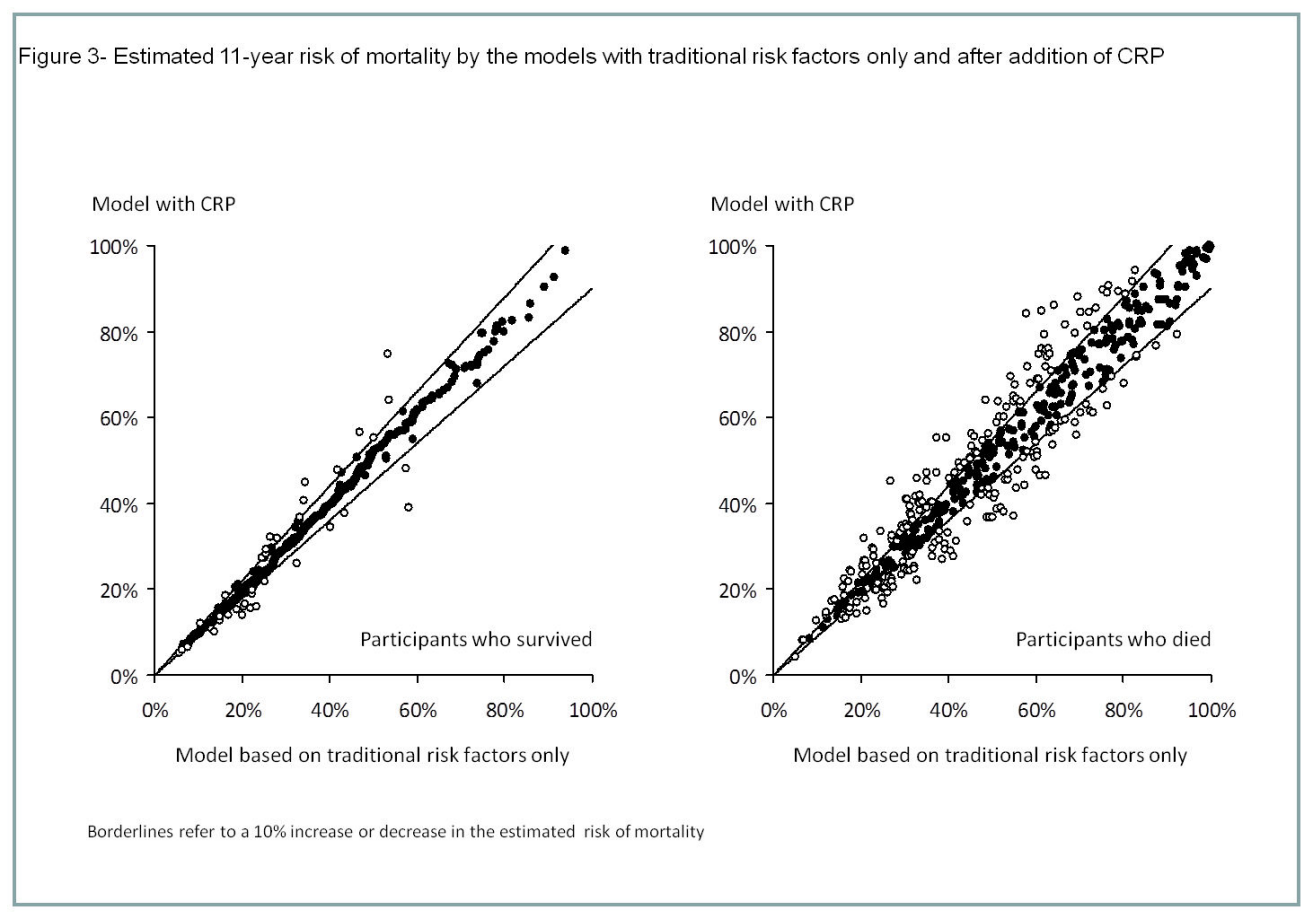
Estimated 11-year risk of mortality by the models with traditional risk factors only and after addition of CRP.

**Figure 4:**
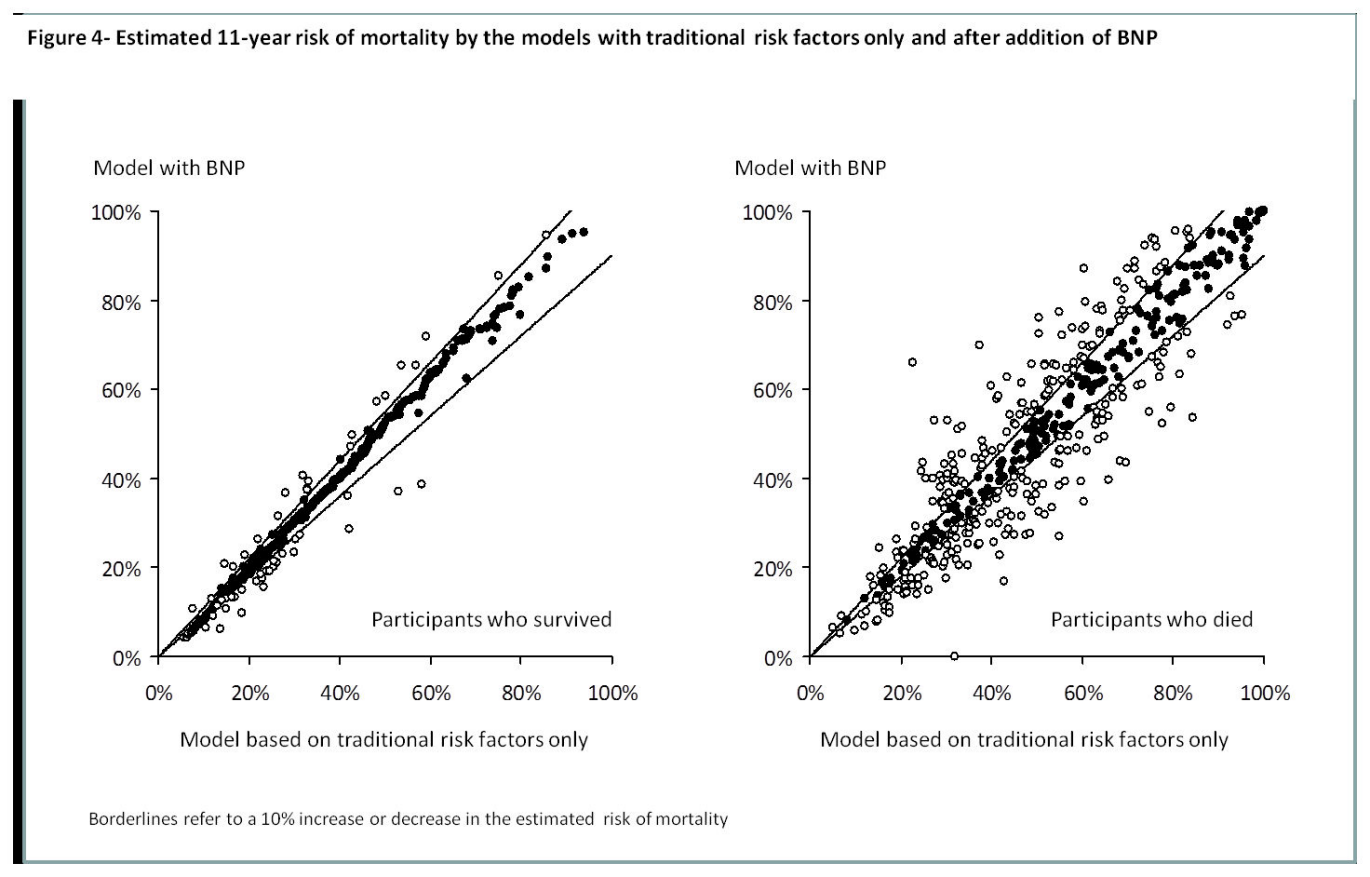
Estimated 11-year risk of mortality by the models with traditional risk factors only and after addition of BNP.

**Figure 5:**
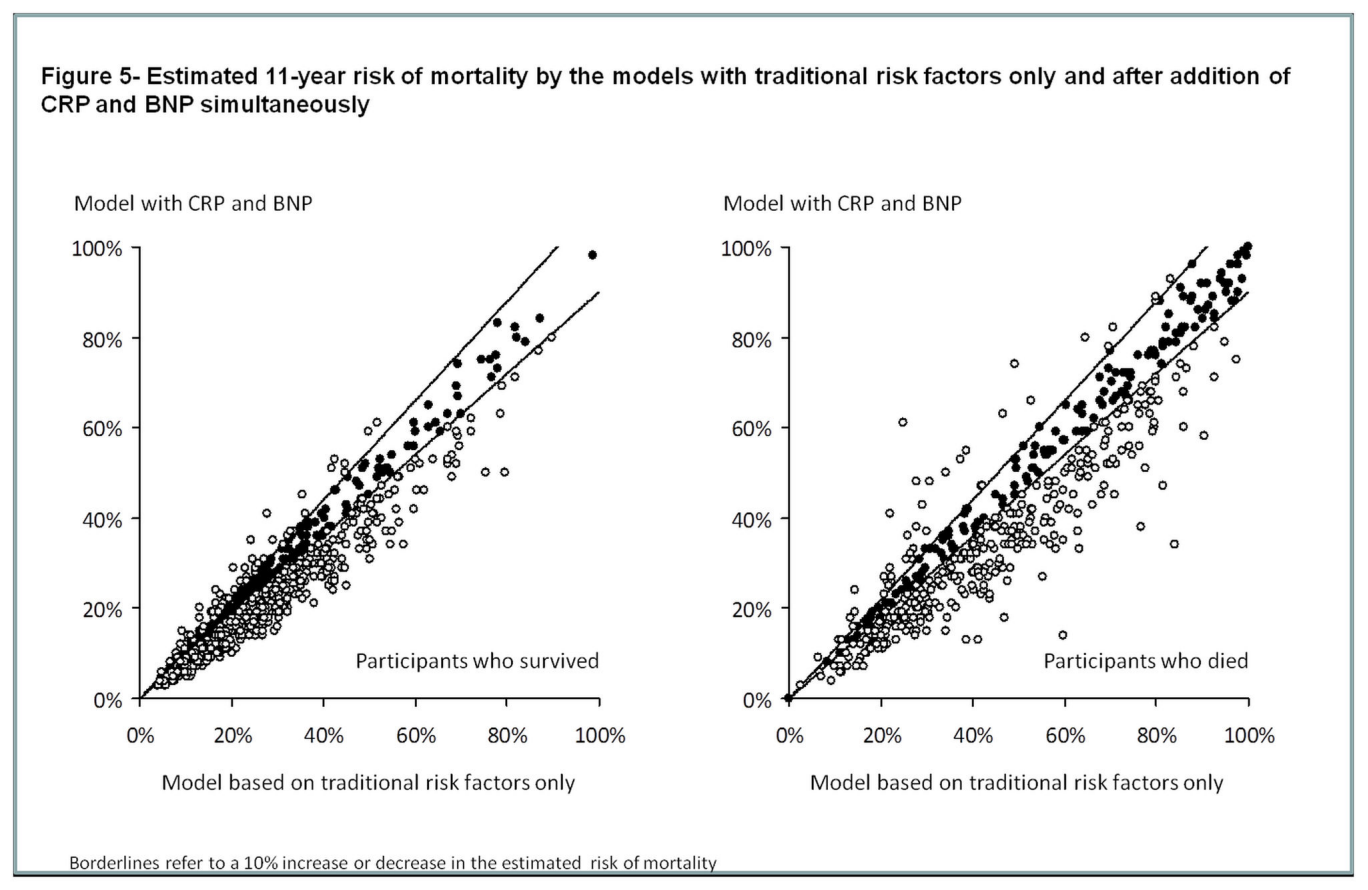
Estimated 11-year risk of mortality by the models with traditional risk factors only and after addition of CRP and BNP simultaneously.

IDI was modest for the addition of CRP (IDI=0.009; p=<0.001), BNP (IDI=-0.005; p=<0.001) and both of the biomarkes (IDI=-0.003; p=0.84) to the model with traditional risk factors only. Moreover, the addition of CRP led to small non-significant changes in the prediction of death (NRI=0.04; 95% CI -0.02 to 0.09; p=0.24) by the model. Changes in the risk of death estimated by the model were also non-significant when BNP was added to traditional risk factors (NRI=0.07; 95% CI -0.01 to 0.14; p=0.08) and when BNP and CRP were simultaneously added to the model (NRI=0.06; 95% CI -0.01 to 0.12; p=0.10) Figures 3-5 display the estimated risk of death for both subjects who died and survived during the follow-up time for each of the new models compared to the model based on traditional risk factors only.

## Discussion

In a population of community-dwelling elderly in Bambuí, Brazil, CRP and BNP were determinants of long-term mortality independently of traditional risk factors. However, although the models with the biomarkers were well-calibrated, neither CRP nor BNP individually or in combination led to a significant improvement in the ability of the model in discriminating different levels of risk of mortality. Furthermore, the addition of the biomarkers led to at most small correct changes in the estimation of the risk of mortality. This suggests that, although there is a clinical demand to predict death in the elderly to aid in decisions regarding preventative and curative interventions as well as palliative care, the incremental value of CRP and/or BNP compared to more established risk factors is probably too modest.

### Biological explanations

CRP and BNP are components of different biological pathways that may lead to death. The positive association between CRP and increased mortality in the elderly may be directly associated with the physiological immune response associated with the ageing process [29] or be mediated by a broad range of inflammatory and thrombotic conditions, such as atherosclerosis [30] and cardiovascular diseases [31,32]. As well, augmented hs-CRP levels are associated with an increased risk of decreased muscle function [33], disability and functional decline [34], osteoporotic and non-traumatic fractures [35,36], visceral adiposity [37], and hospitalization [38].

Several biological mechanisms are implicated in the association between high BNP levels and death in the elderly. Systolic and diastolic dysfunction can lead to an increase in plasmatic BNP levels. Myocardial fibrosis, ventricular hyperthophy, preclinical and symptomatic myocardial ischemia, and renal dysfunction, which are associated with an increased risk of death in the elderly, lead to elevated BNP levels [39]. Moreover, due to still poorly defined mechanisms, high BNP levels are associated with a higher incidence of stroke [40], as well as with poor functional outcomes [41]. In our population, Chagas disease, which is independently associated with high BNP levels [42], is an additional factor to explain the increased risk of death related to elevated BNP levels.

### Comparison with other studies

Previous studies that investigated the issue of the value of hs-CRP in predicting cause-specific, mostly cardiovascular, and/or overall mortality in community-dwelling elderly yielded heterogeneous results. Neither was hs-CRP independently related to overall mortality in an older adult population without heart or renal failure followed-up for five years [39] nor in women aged at least 65 years old [43]. Moreover, it did not predict the combined end-point of major CV events and CV death in a population of older adults followed-up for approximately 10 years [44]. On the other hand, similarly to our findings, CRP was an independent determinant of 4-year all-cause mortality both in community-dwelling populations of frail [4] and non-disabled [5] elderly.

Regarding the association between natriuretic peptides and mortality, our results point to an independent prognostic value of BNP, in agreement with other population-based studies which investigated the role of different NPs as determinants of overall and cause-specific death in older adults [10] [45] [46], including a previous investigation in the elderly with Chagas disease in the BHAS [47]. However, none of these studies with CRP or BNP performed measures of the incremental value of the biomarker in predicting overall mortality.

Regarding the association of CRP and BNP as prognostic markers, an investigation of the Framingham Offspring Study which compared C-statistic between models with traditional risk factors only and with the addition of a multi-marker score that included both hs-CRP and BNP found that only BNP led to a to a small increase in the ability of the model in discriminating between death and survival in older adults [48]. On the other hand, CRP and NT-proBNP, a precursor of BNP, were independently related to CV and overall mortality and a score formed by the two biomarkers plus three others substantially improved the predictive ability over conventional risk factors in a population of older adults with mean age of 71 years [49].

### Strengths and limitations

Comparing different aspects of the models predictive performance by NRI and IDI, and not only by hazard ratios and discrimination, which have been shown to have important pitfalls [50], strengthen the results of the present investigation. The long-term follow-up, the minimal lost to follow-up, and adjustment for a large set of potential confounders on the association between the biomarkers and death are other major strengths of our study. Particularly, adjustment for WC, which is a surrogate of visceral adiposity, can be important, as previous studies observed that the age-associated variation in CRP is related to the amount of visceral adipose tissue [37], and that visceral adiposity predicts death. We believe that these results can be generalized to other Brazilian populations with a high prevalence of cardiovascular diseases.

However, several limitations must be acknowledged. We only had a single baseline measurement of each of the biomarkers. Regarding hs-CRP, an extra measurement can be advisable due to fluctuations in systemic inflammatory status over time [16]. We were not able to investigate the influence of medications, such as statins, anti-inflammatory and anti-diabetic drugs, on hs-CRP levels. Whether the prognostic value of the biomarkers differs between cause-specific and overall mortality was not addressed in the present study either.

In conclusion, CRP and BNP individually or combined are independent determinants of death in this community-dwelling elderly population. However, the incremental value of these biomarkers to traditional risk factors seems to be either small or non-significant. These findings point out that CRP and BNP probably should not be used to aid in decisions about prevention of overall mortality in the elderly. Further investigation of cost-benefit issues is also necessary to better understand the incremental value of these biomarkers.
